# The earliest fossil record of Panorpidae (Mecoptera) from the Middle Jurassic of China

**DOI:** 10.3897/zookeys.431.7561

**Published:** 2014-08-06

**Authors:** He Ding, Chungkun Shih, Alexei Bashkuev, Yunyun Zhao, Dong Ren

**Affiliations:** 1 Key Lab of Insect Evolution and Environmental Change, College of Life Sciences, Capital Normal University, 105 Xisanhuanbeilu, Beijing 100048, China; 2 Borissiak Paleontological Institute, Russian Academy of Sciences, Profsoyuznaya st. 123, Moscow 117997, Russia

**Keywords:** *Jurassipanorpa*, new genus, new species, new species, Jiulongshan Formation, Daohugou

## Abstract

The early history of Panorpidae (Mecoptera) is poorly known due to sparse fossil records. Up to date, only nine fossil species have been described, all from the Paleogene, except the Early Cretaceous *Solusipanorpa gibbidorsa* Lin, 1980. However, we suggest *S. gibbidorsa* is too incompletely preserved to permit even family classification. A new genus with two new species, *Jurassipanorpa impunctata* **gen. et sp. n.** and *Jurassipanorpa sticta* **sp. n.**, are described based on four well-preserved specimens from the late Middle Jurassic Jiulongshan Formation of Daohugou, Inner Mongolia, China. These two new species are the earliest fossil records of Panorpidae. The new genus is erected based on a combination of forewing characters: both R_1_ and Rs_1_ with two branches, 1A reaching posterior margin of wing distad of the forking of Rs from R_1,_ and no crossveins or only one crossvein between veins of 1A and 2A. In all four specimens, long and robust setae ranging from 0.09 to 0.38 mm in length and pointing anteriorly, are present on anal veins of forewings. The function of these setae is enigmatic.

## Introduction

Mecoptera is a small order of insects, comprising about 600 extant species assigned to nine families (Cai et al. 2008, Krzemiński and Soszyńska-Maj 2012). The Panorpidae is the largest family in the order, with about 400 extant species in six genera: *Panorpa* Linnaeus, 1758, *Leptopanorpa* MacLachlan, 1875, *Neopanorpa* Weele, 1909, *Sinopanorpa* Cai, Huang & Hua, 2008, *Furcatopanorpa* Ma & Hua, 2011 and *Dicerapanorpa* Zhong & Hua, 2013 (Byers 1967, Cai et al. 2008, Cai and Hua 2009, Ma and Hua 2011, Zhong and Hua 2013).

Fossil records of the Panorpidae are fairly rare. Up to date, only three genera have been described in this family and two of them are fossil-only genera: *Solusipanorpa* Lin, 1980 with one species from the Early Cretaceous of China (Lin 1980); *Baltipanorpa* Krzemiński, 2012 with one species from the Eocene Baltic amber (Krzemiński and Soszyńska-Maj 2012); and *Panorpa* Linnaeus, 1758 with seven species from Baltic amber, Eocene of U.S.A. and Oligocene of Germany (Carpenter 1931, Statz 1936, Carpenter 1954). However, the holotype (and the only known specimen) of *Solusipanorpa gibbidorsa* is too incompletely preserved and cannot be attributed to any particular family. In addition, Archibald et al. (2013: fig. 23) reported several undescribed specimens of Panorpidae in the Early Eocene Okanagan Highlands, Canada and U.S.A. Therefore, with *Solusipanorpa gibbidorsa* ignored, the fossil records of Panorpidae are known so far since the Early Eocene.

Recently we collected four well-preserved fossils, which we attribute to Panorpidae, from the Jiulongshan Formation at Daohugou, Ningcheng County, Inner Mongolia, China. The Jiulongshan Formation is dated as the late Middle Jurassic, ca. 165 Ma (Ren et al. 1995, 2002, Shen et al. 2003, Chen et al. 2004, Liu et al. 2004, Ji et al. 2006, Gao and Ren 2006, Huang et al. 2006, Wang et al. 2013). Based on a combination of forewing characters: both R_1_ and Rs_1_ with two branches, 1A reaching posterior margin of wing distad of the forking of Rs from R_1,_ and no crossveins or only one crossvein between veins 1A and 2A, we erect a new genus *Jurassipanorpa* with two new species.

## Materials and methods

This study is based on four fossil specimens collected from the late Middle Jurassic Jiulongshan Formation at Daohugou Village of Ningcheng County in Inner Mongolia, China. All type specimens are housed in the fossil insect collection of the Key Laboratory of Insect Evolution & Environmental Changes, College of Life Sciences, Capital Normal University, Beijing, China (CNUB; Dong Ren, Curator).

The specimens were examined and photographed using a Leica MZ12.5 dissecting microscope with a Leica DFC 500 digital camera and illustrated with the aid of a drawing tube attachment. The line drawings were drawn by Adobe Photoshop CS5. We use the venational nomenclature of Willmann (1989).

## Systematic Palaeontology

### Order Mecoptera Packard, 1886
Family Panorpidae Latreille, 1805

#### Jurassipanorpa

Taxon classificationAnimaliaMecopteraPanorpidae

Ding, Shih & Ren gen. n.

http://zoobank.org/5D919FDF-18E3-48D5-B723-2BDCE98B900B

##### Etymology.

The generic name is a combination of Jurassic, highlighting the age of these fossil panorpids, and *Panorpa*, the type genus of Panorpidae. Gender feminine.

##### Type species.

*Jurassipanorpa impunctata* Ding, Shih & Ren, sp. n.

##### Other included species.

*Jurassipanorpa sticta* Ding, Shih & Ren sp. n.

##### Diagnosis.

In forewing, Sc reaching the anterior margin near or beyond the middle of the wing; both R_1_ and Rs_1_ with two branches; 1A reaching posterior margin distad of the forking of Rs from R_1_; 3 anal veins present; one crossvein between Cu_1_ and Cu_2_ and between 1A and 2A respectively.

##### Remarks.

We assigned this genus to Panorpidae mainly based on the following characters: (1) head capsule with prolonged downward mouthparts; (2) slender wings and forewing slightly larger than hind wing with similar veins; (3) forewing Rs with five branches; (4) forewing M with four branches; (5) forewing Cu_1_ not fused with M basally, one crossvein between M_4_ and Cu_1_; and (6) hind wing Rs with five and M with four branches as those of forewing.

#### Jurassipanorpa impunctata

Taxon classificationAnimaliaMecopteraPanorpidae

Ding, Shih & Ren sp. n.

http://zoobank.org/021EE774-F1FC-4C91-A91F-7E39A5B82809

Figs 1 , 2

##### Etymology.

From the Latin *impunctata*, meaning no spots, referring to the fact that no spots and fasciae on all wings.

##### Holotype.

CNU-MEC-NN-2013006, a well-preserved female specimen with body and wings, but legs poorly preserved.

##### Paratype.

CNU-MEC-NN-2013012 P/C, sex unknown, with well-preserved legs, but four wings overlapping almost entirely and abdomen partially preserved.

##### Locality and horizon.

Jiulongshan Formation, late Middle Jurassic; Daohugou Village, Ningcheng County, Inner Mongolia, China.

##### Diagnosis.

On both fore- and hind wings, Rs_1+2_ shorter than Rs_1a+1b_, Rs_1+2_ shorter than Rs_3+4_, Rs and M forking at the same level and no spots or fasciae.

##### Description.

**Figure 1.**
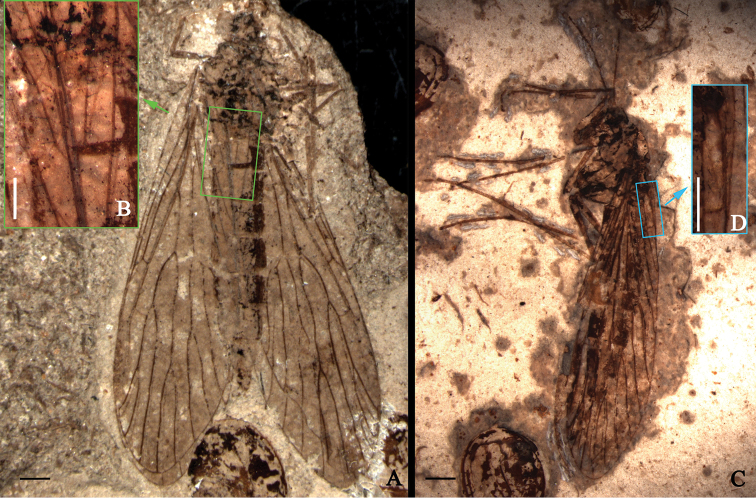
*Jurassipanorpa impunctata* gen. et sp. n., holotype, CNU-MEC-NN-2013006; paratype, CNU-MEC-NN-2013012 P/C. photos. **A** holotype **B** setae on forewings, under alcohol, outlined at rectangular frame in **A**; **C** paratype, under alcohol **D** setae on forewings, under alcohol, outlined at rectangular frame in **C.** Scale bars: 1 mm in **A**; 0.5 mm in **B, C**; 2 mm in **C.**

**Figure 2.**
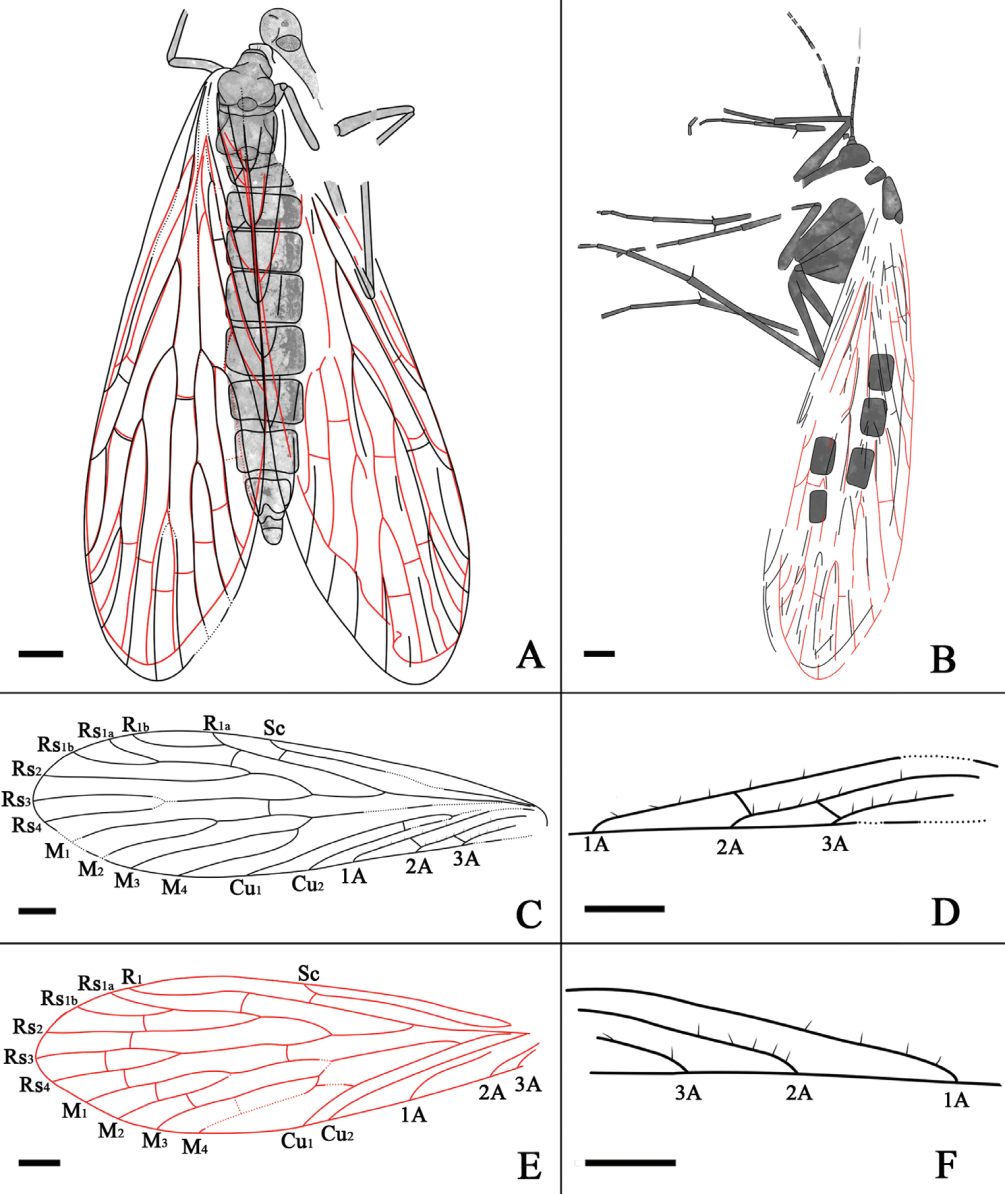
*Jurassipanorpa impunctata* gen. et sp. n. line drawings. **A** holotype **B** paratype **C** left forewing of the holotype **D** anal part of left forewing highlighting setae of the holotype **E** left hind wing of the holotype, crossvein between M_3_ and M_4_ is based on the right hind wing **F** right forewing highlighting setae of the holotype. Scale bars: 1 mm in **A–F.**

Mainly based on Holotype, unless indicated as paratype. A well-preserved female adult fossil. Body 12 mm long. Forewing and hind wing overlapping almost entirely, but most of veins discernible. Thorax and abdomen preserved, but head poorly preserved. Legs poorly preserved, with only few fragments (Figs 1A, 2A, 2C–F).

*Head*: Head capsule with downward extended mouthparts; compound eyes large and oval, three ocelli present (Figs 1A, 2A); Antennae filiform in paratype (Figs 1C, 2B).

*Thorax*: In dorsal view, 2.9 mm long, two setae on pronotum as preserved. Pronotum, mesonotum, metanotum clearly discernible. Meso- and metanotum about the same size; larger than pronotum.

*Abdomen*: In dorsal view, 9 mm long, tapering apically, with eleven visible segments; segments IX-XI more slender and shorter than segments II-VI. Cerci not preserved. Sterna visible in segments II-VI.

*Legs*: Densely covered with short setae, two long tibial spurs preserved in a mid leg, one tibial spur preserved in a fore leg and two hind legs in paratype (Figs 1C, 2B).

*Wings*: Venation similar to venation of *Panorpa*. Forewing (Figs 1A, 2A, B, C, D, F) 14 mm long with a maximal width of 4 mm, longer than the abdomen; Sc terminating at anterior margin near the middle of the wing; one distally located crossvein between Sc and R_1_; R_1_ long, branching and curving around pterostigma; one crossvein present between R_1_ and Rs_1_; Rs with five branches; Rs_1_ forking into Rs_1a_ and Rs_1b_; Rs_1_ and Rs_3+4_ forking nearly at the same level; Rs_1+2_ forking proximad of Rs_3+4_ forking; one crossvein between Rs_3+4_ and M_1+2_; Rs and M forking at the same level; M with four branches; M_3+4_ shorter than M_1+2_; Cu_1_ not fusing with M basally; one crossvein present between Cu_1_ and Cu_2_; 1A long, reaching posterior wing margin beyond the forking of Rs from R_1_; one crossvein between 1A and 2A; 2A and 3A long, one crossvein between 2A and 3A; long and robust setae ranging from 0.09 to 0.17 mm in length, present on veins 1A, 2A and 3A (Figs 1B, D, 2D, F). Hind wing (Figs 1A, 2A, E), 12.3 mm long with a maximal width of 3.9 mm, smaller than forewing distinctly, of similar shape and veins; Sc short, reaching anterior wing margin before one-half wing length; one crossvein present between Sc and R_1_; R_1_ without forking; Rs with five branches; Rs and M forking at almost the same level; one crossvein present between R_1_ and Rs_1_; Rs_1_ forking into Rs_1a_ and Rs_1b_; one crossvein between Rs_1b_ and Rs_2_; Rs_2_ without forking; one crossvein between Rs_2_ and Rs_3_; Rs_1+2_ forking proximad of Rs_3+4_ forking; one crossvein between Rs_3_ and Rs_4_; M_1+2_ forking proximad of Rs_3+4_ forking; one crossvein between Rs_3+4_ and M_1+2_ and one crossvein between Rs_4_ and M_1_; M with four branches; one crossvein between M_1_ and M_2_; M_3+4_ shorter than M_1+2_; one crossvein between M_2_ and M_3_ and between M_3_ and M_4_ respectively; Cu_1_ coalesced with M basally; one crossvein between M_4_ and Cu_1_; no crossveins between anal veins.

**Figure 3.**
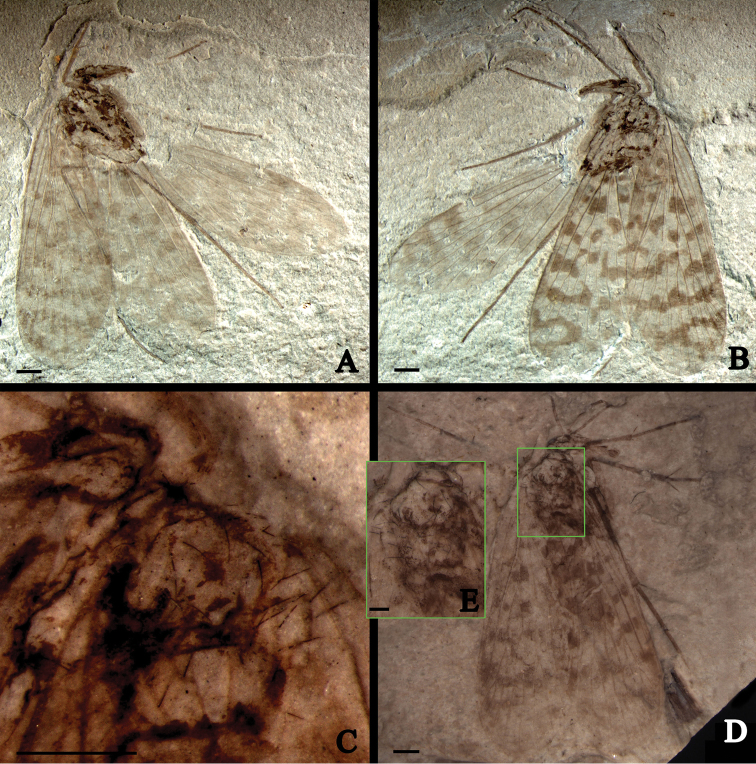
*Jurassipanorpa sticta* sp. n., holotype, CNU-MEC-NN-2013007 P/C; paratype, CNU-MEC-NN-2013011. photos. **A** part of the holotype **B** counterpart of the holotype **C** anal part of forewings of the holotype, under alcohol **D** paratype, under alcohol **E** setae on forewings, under alcohol, outlined at rectangular frame in D. Scale bars: 1 mm in A–D, 0.5 mm in **E.**

#### Jurassipanorpa sticta

Taxon classificationAnimaliaMecopteraPanorpidae

Ding, Shih & Ren sp. n.

http://zoobank.org/73034C76-F459-48E5-A12C-EA1989478855

Figs 3 , 4

##### Etymology.

From the Greek *stiktos*, meaning spotted, referring to various spots and fasciae on wings.

##### Holotype.

CNU-MEC-NN-2013007 P/C, part and counterpart, sex unknown, well- preserved fore- and hind wings, but abdomen indiscernible.

##### Paratype.

CNU-MEC-NN-2013011, sex unknown, anal part of forewings well- persevered.

##### Locality and horizon.

Jiulongshan Formation, late Middle Jurassic; Daohugou Village, Ningcheng County, Inner Mongolia, China.

##### Diagnosis.

On both fore- and hind wings, Rs_1+2_ longer than Rs_1a+1b_ and Rs_1+2_ longer than Rs_3+4_. Rs and M forking at the same level on forewing but Rs forking proximad of M forking on hind wing. All wings with scattered dark spots and fasciae.

##### Description.

**Figure 4.**
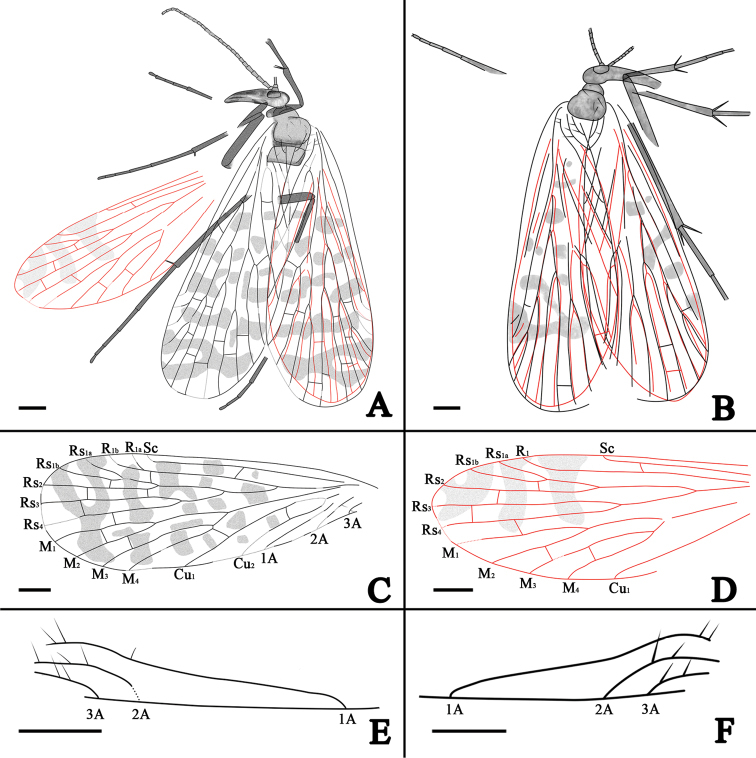
*Jurassipanorpa sticta* sp. n. line drawings. **A** holotype **B** paratype **C** left forewing of the holotype **D** left hind wing of the holotype **E** anal part of right forewing highlighting setae of the holotype **F** left forewing highlighting setae of the paratype. Scale bars: 1 mm in **A–F.**

Mainly based on Holotype, unless indicated as paratype. A well-preserved adult fossil, sex unknown. Right forewing and hind wing nearly overlapping entirely, but most veins discernible, left forewing and right wings partially overlapping, left hind wing well preserved (Figs 3A, B, 4A).

*Head*: Head capsule with prolonged downward mouthparts as modern panorpids. Compound eyes large and oval; ocelli untraceable; antennae filiform, with 25 segments as preserved.

*Thorax*: poorly preserved (Figs 3A–C, 4A), only prothorax and part mesothorax recognizable. Few setae on tergum.

*Legs*: Densely covered with short setae; one mid leg not preserved; coxae and trochanters of all legs not preserved; femur and tibia long; two long tibial spurs present on a fore leg, one tibial spur present on a hind leg and one of them incomplete on a mid leg.

*Abdomen*: Indiscernible.

*Wings*: Forewing, 11 mm long with a maximum width of 3.9 mm, with scattered dark spots and fasciae (Figs 3A–C, 4A, C, E) with a different pattern from those of extant Panorpidae. Sc long, reaching anterior wing margin beyond one-half of wing length; one crossvein between Sc and R_1_ located nearly of mid-length of Sc; R_1_ long with two branches; one crossvein between R_1_ and Rs_1_; Rs with five branches, originating from R_1_ nearly basal 1/3 of forewing length; Rs_1_ forking into Rs_1a_ and Rs_1b_; one crossvein between Rs_1b_ and Rs_2_; two crossveins between Rs_2_ and Rs_3_; Rs_1+2_ forking distad to Rs_3+4_ forking; one crossvein between Rs_3_ and Rs_4_; Rs and M forking at almost the same level; one crossvein between Rs_4_ and M_1_; M with four branches; one crossvein between M_1_ and M_2_; M_3+4_ shorter than M_1+2_; one crossvein between M_2_ and M_3_; Cu_1_ not fusing with M basally, but joining M_4_ by a oblique crossvein; Cu_1_ fusing with Cu_2_ basally and one crossvein between Cu_1_ and Cu_2_; 1A long, reaching posterior wing margin distad to the origination of Rs from R_1_; 2A and 3A short; long and robust setae ranging from 0.15 to 0.38 mm in length, present on veins 1A, 2A and 3A of holotype and paratype (Fig. 4E, F). Left hind wing extended, right hind wing covered by the right forewing, incomplete; hind wing 9 mm long with a maximal width of 3.2 mm, smaller than forewing distinctly, of similar shape and veins (Figs 3A, B, 4A, D); but fasciae much reduced. Sc short, reaching anterior wing margin beyond one-half of wing length; one crossvein between Sc and R_1_; R_1_ long without forking; Rs with five branches; Rs_1_ forking into Rs_1a_ and Rs_1b_; one crossvein between Rs_1b_ and Rs_2_; Rs_1_ shorter than Rs_1+2_; one crossvein between Rs_2_ and Rs_3_; Rs_1+2_ forking distad to Rs_3+4_ forking; one crossvein between Rs_3_ and Rs_4_; M furcating distad to the Rs forking; one crossvein between Rs_4_ and M_1_; M with four branches; one crossvein between M_2_ and M_3_; M_3+4_ shorter than M_1+2_; one crossvein between M_3_ and M_4_; Cu_1_ coalesced with M basally; no crossveins present between M and Cu_1_; anal veins not discernible on hind wing due to poor preservation.

##### Comparison.

*Jurassipanorpa sticta* sp. n. is differentiated from *Jurassipanorpa impunctata* gen. et sp. n. by the following characters: (1) *Jurassipanorpa sticta* with various spots and fasciae (vs. *Jurassipanorpa impunctata* without spots and fasciae); (2) Rs_1+2_ longer than Rs_1a+1b_ (vs. Rs_1+2_ shorter than Rs_1a+1b_); (3) Rs_1+2_ longer than Rs_3+4_ (vs. Rs_1+2_ shorter than Rs_3+4_) (4) Rs and M forking at the same level on forewing, but Rs furcating proximad of M forking on hind wing (vs. Rs and M forking at the same level on both fore- and hind wing).

## Discussion

In the vast insect fossil collection at the Capital Normal University (> 250,000 fossil insect specimens), we have collected only four panorpid fossils so far from the Daohugou locality. Informal survey of the Mecoptera collection indicates that specimens of Nannochoristidae are abundant, followed by many specimens of Orthophlebiidae, Bittacidae, and Cimbrophlebiidae, then, low numbers of Choristopsychidae, Mesopsychidae, Aneuretopsychidae, Pseudopolycentropodidae, and Eomeropidae, while very rare for Panorpidae. It is interesting to note that the rarity of Panorpidae during the Middle Jurassic of northeastern China is in contrast to their dominance in the Recent world fauna of Mecoptera (about 66% of all extant species).

Described fossil records of the Panorpidae in Mesozoic are extremely rare. Up to now, only one species, *Solusipanorpa gibbidorsa* Lin, 1980, has been described. However, we consider the holotype of *Solusipanorpa gibbidorsa* as not sufficiently preserved to be attributed to Panorpidae nor to any other family, and regard *Solusipanorpa gibbidorsa* as Mecoptera incertae sedis. Eight species in two genera: *Panorpa* Linnaeus, 1758, and *Baltipanorpa* Krzemiński, 2012, have been reported from the Eocene and Oligocene, however Carpenter stated that “*Panorpa rigida* Scudder, from the Florissant shales, is too incompletely preserved to permit even family classification.” and “*Panorpa arctiiformis* Cockerell, also from the Florissant shales, is undoubtedly a member of the family Panorpidae, but I have not seen the type specimen and there is nothing in the description to indicate its affinities.” (Carpenter 1931, Carpenter 1954, Willmann 1989, Krzemiński and Soszyńska-Maj 2012, Archibald et al. 2013). *Jurassipanorpa impunctata* gen. et sp. n. and *Jurassipanorpa sticta* sp. n. described in this study are the earliest fossil panorpids in the world hitherto. The holotypes of these two new species of *Jurassipanorpa* gen. n. are well-preserved, including both wings and most of the body. New information from these two new species enhances our understanding of the morphological characters of Panorpidae and diversity of Mecoptera during the late Middle Jurassic.

Based on studies of these two new species and documented species of other representative panorpids, we compare and summarize six key forewing characters in Table 1. A combination of the following characters enables us to distinguish the new genus from all other described genera of Panorpidae: (1) R_1_ with two branches in forewing – same as *Baltipanorpa*, *Furcatopanorpa* and a rare few species of *Panorpa*, but different from all other genera with only one branch; (2) Rs_1_ with two branches of Rs_1a_ and Rs_1b_ – different from *Sinopanorpa* with 3 branches; (3) 1A reaching posterior margin distad of the forking of Rs from R_1_ in forewing – same as *Panorpa*, *Sinopanorpa*, *Furcatopanorpa*, *Dicerapanorpa*, and *Baltipanorpa*, in contrast to significantly shortened 1A in *Neopanorpa* and *Leptopanorpa*; (4) no crossveins or only one crossvein between 1A and 2A on forewing – different from almost all other genera of Panorpidae, *Neopanorpa* and *Leptopanorpa* (one crossvein), *Panorpa* (one or two crossveins), *Sinopanorpa*, and *Dicerapanorpa* (two crossveins), *Baltipanorpa* and *Furcatopanorpa* (three crossveins); (Byers 1967, Cai et al. 2008, Ma and Hua 2011, Krzemiński and Soszyńska-Maj 2012, Zhong and Hua 2013).

We found that the wing venation of this family is comparatively stable, that is, Sc not forking, Rs_2_, Rs_3_, Rs_4_ without forking, and M with 4 branches. However, Rs_1_ is rather variable, typically, Rs_1_ has two branches, but, some with three branches such as *Sinopanorpa*, while three Mexican extant species *Panorpa dividilacinia, P. mixteca, P. umbricola* have only a single Rs_1_ (Carpenter 1992, Bicha 2006, Cai et al. 2008).

Most panorpids have M_4_ bending basally in forewings, that is, M_3_ is a straight branch while M_4_ is derived from M_3_ with a basal bending. However, this character is not obvious for some extant panorpids, such as *Panorpa wangwushana* Huang, Hua and Shen, 2004 (Huang et al. 2004). On the other hand, most panorpodids have M_3_ bending basally in forewings, that is, M_4_ is a straight branch while M_3_ is derived from M_4_ with a basal bending. However, *Panorpodes colei* Byers, 2005 is an exception to this typical panorpodid character (Byers 2005). *Jurassipanorpa impunctata* gen. et sp. n. has M_4_ bending basally in forewing (Figs 2A, C), but *Jurassipanorpa sticta* sp. n. does not have obvious M_4_ bending basally, nor M_3_ bending basally (Figs 4A, C). Hence, we propose that M_4_ or M_3_ basal bending is variable in both Panorpidae and Panorpodidae.

All four specimens of *Jurassipanorpa impunctata* gen. et sp. n. (Figs 1B, D, 2D, F) and *Jurassipanorpa sticta* sp. n. (Figs 3C, E, 4E, F) have remarkable long and robust setae on anal veins of the forewings that are not known for any other described fossil or extant panorpid species. Unlike these of *Jurassipanorpa* gen. n., setae on veins of extant species of Panorpidae as well as of Mesozoic Orthophlebiidae are usually more dense, shorter and decumbent unidirectionally and look similar to microtrichia on the wing membrane (Tillyard 1933, Webb and Penny 1979, Whalley 1985, Whalley 1986). These setae of the new genus, with lengths ranging from 0.09 to 0.38 mm, are similar in appearance to the piliform scales, ranging from 0.12 to 0.21 mm in lengths, present on the hind wing veins of *Akainalepidopteron elachipteron* Zhang, Shih, Labandeira & Ren, 2013 of Lepidoptera (Zhang et al. 2013), or to robust bristles (“dinotrichia”) on the basal anterior margin of forewings on the Recent *Notiothauma reedi* and fossil *Tsuchingothauma shihi* and *Jurathauma simplex* (Eomeropidae) (Crampton 1930, Ren and Shih 2005, Zhang et al. 2011). The wing scales have been used as a diagnostic character for Lepidoptera, while also found in some forewings of Trichoptera (Kristensen 1984, Grimaldi 1999, Zhang et al. 2013). However, it is unlikely that these setae are homologous to piliform scales because scales have not been reported for Panorpidae. Since these setae are located only on anal veins of the forewings while all pointing anteriorly, we hypothesized that they might have been used for wing coupling. But, we could not find any associated structures preserved on the anterior part of the hind wings on these specimens. Hence, the function of these setae remains enigmatic.

**Table 1.** Comparison of fossil and extant genera of Panorpidae with six key forewing characters.

	**Genus**	**Species**	**Position of Sc reaching anterior margin**	**Branches of R_1_**	**Branches of Rs_1_**	**position of 1A reaching the posterior margin**	**number of crossveins between 1A and 2A**	**number of crossveins on forewing**	**Comments**
extant genera	*Panorpa* Linnaeus, 1758	*Panorpa communis* L. 1758	beyond the middle of wing	1 (2 only in rare cases)	2–3	far beyond the forking of Rs from R_1_	2	about 22	
	*Neopanorpa* Weele, 1909	*Neopanorpa appendiculata* (Westwood, 1846)	beyond the middle of wing	1	2	not beyond the forking of Rs from R_1_	1	about 23	
	*Leptopanorpa* MacLachlan, 1875	*Leptopanorpa ritsemae* MacLachlan, 1875	beyond the middle of wing	1	2	at same level as the forking of Rs from R_1_	1	about 27	wings are slender and much narrower basally
	*Sinopanorp* a Cai and Hua, 2008	*Sinopanorp tincta* (Navas, 1931)	beyond the middle of wing	1	3	far beyond the forking of Rs from R_1_	2	about 22	
	*Furcatopanorpa* Ma & Hua, 2011	*Furcatopanorpa longihypovalva* (Hua & Cai, 2009)	near the middle of wing	2 (fore- and hind wings)	2	far beyond the forking of Rs from R_1_	3	about 26	wings held roof-like over the abdomen at rest.
	*Dicerapanorpa* Zhong & Hua, 2013	*Dicerapanorpa magna* (Chou, 1981)	beyond the middle of wing	1 (but 2 in hind wings of some cases)	2	far beyond the forking of Rs from R_1_	2	about 26	
fossil genera	*Baltipanorpa* Krzemiński, 2012, Eocene	*Baltipanorpa damzeni* Krzemiński, 2012	before the middle of wing	2	2	at same level as the forking of Rs from R_1_	3	about 26	
	*Jurassipanorpa* gen. n., Middle Jurassic	*Jurassipanorpa impunctata* gen. et sp. n.	at the middle of wing	2	2	far beyond the forking of Rs from R_1_	1	6	no spots and fasciae
		*Jurassipanorpa sticta* sp. n.	beyond the middle of wing	2	2	far beyond the forking of Rs from R_1_	0	11	spots and fasciae much different from extant Panorpidae

## Supplementary Material

XML Treatment for Jurassipanorpa

XML Treatment for Jurassipanorpa impunctata

XML Treatment for Jurassipanorpa sticta
